# A Longitudinal Study of Cultural Consonance, Personal Agency, and Psychological Distress in Urban Brazil

**DOI:** 10.3390/bs14090762

**Published:** 2024-08-30

**Authors:** William W. Dressler, Mauro C. Balieiro, José Ernesto dos Santos

**Affiliations:** 1 Department of Anthropology, The University of Alabama, Tuscaloosa, AL 35401, USA; 2 Department of Psychology, Paulista University, Ribeirão Preto 05347-020, SP, Brazil; maurobalieiro@gmail.com; 3 Department of Internal Medicine, Faculty of Medicine, University of São Paulo—Ribeirão Preto Ribeirão, Preto 13635-900, SP, Brazil; jedsanto@fmrp.usp.br

**Keywords:** cultural consonance, personal agency, psychological distress, Brazil

## Abstract

The relationship between culture, as a set of norms that structure human social practice, and agency, as the human capacity to act, has been debated for decades. Achieving clarity in how these constructs intersect has been hampered by difficulty in measuring either one, and theory has not suggested how a model linking culture and agency might be specified. We present a model in which culture is measured as cultural consonance, or the degree to which individuals actually incorporate prototypes for behavior encoded in cultural models into their own behavior. This measurement is then integrated with a measure of individuals’ sense of personal agency. In a previous study in urban Brazil, we found that personal agency was associated with higher cultural consonance, which in turn was associated with lower psychological distress; however, those data were from a cross-sectional survey, thus limiting the causal inference. Here we present the results of a follow-up study in which a subset of respondents was re-interviewed on average four years later. These data are consistent with a model in which cultural consonance is the proximate causal influence on psychological distress, while personal agency is a distal or exogenous influence. The implications of these results for the relative roles of culture and agency as influences on subjective well-being are discussed.

## 1. Introduction

The role of the individual in sociocultural processes has been debated throughout the history of the social sciences, some even arguing that it is the key theoretical dilemma of the past century [1]. The theoretical conundrum has often been referred to as the opposition between structure and agency [2], which has occupied the attention of some of the luminaries of social scientific theory of the last fifty years [1,3,4,5].

In anthropology, this discussion has revolved around culture and agency, and especially the degree to which individuals are capable of being independent, acting agents under the directive and structuring influence of culture [6]. Put simply, are we “cultural dopes”, merely re-enacting the guiding forces of cultural norms and expectations, or are we independent agents pursuing our own goals regardless of—indeed, in spite of—the structuring influence of culture? Furthermore, what are the relative effects of these two factors on outcomes such as subjective well-being?

Our aim in this paper is to pursue these questions using data collected in urban Brazil. In a previous paper, we presented a test of a causal model in which socioeconomic status and a sense of personal agency were hypothesized to be distal or exogenous variables leading to higher cultural consonance in adult life goals, which measures the degree to which individuals in their own lives match the cultural prototype for life goals. Higher cultural consonance in turn was associated with lower psychological distress [7].

There were several distinctive aspects of this study. First, we operationalized culture using the concept and measurement of cultural consonance [8], thus enabling us to incorporate culture into a causal model and evaluate its causal potential relative to other factors. Second, we operationalized a sense of personal agency, capturing both the dimensions of self-efficacy and the tolerance for the frustration of action. Third, we examined both culture and agency in relation to psychological distress as a measure of subjective well-being, providing an opportunity to contrast the relative influences of both factors on an outcome variable. Finally, the study was carried out in urban Brazil, a cultural context in which both culturally constructed life goals and agency are salient domains in everyday life.

In the following paper, we take this model one step further by testing it longitudinally. We first outline the theory and measurement of our two key constructs—culture and agency. After describing the ethnographic context and research methods in detail, we present a path analysis using longitudinal data that tests the results of the previous cross-sectional study. The implications of these results for the study of culture and agency are then discussed.

Using path analysis, we will test, longitudinally, a model structured by the following hypotheses:

(a) There will be a direct path from socioeconomic status to a sense of personal agency, controlling for Time 1 values of these variables, as well as age and gender.
(b) There will be direct paths from socioeconomic status and personal agency to cultural consonance, controlling for Time 1 values of these variables, as well as age and gender.
(c) There will be an inverse path from cultural consonance to psychological distress, controlling for Time 1 values of these variables, as well as Time 1 and Time 2 values of socioeconomic status and personal agency, and age and gender.

### 1.1. Culture

This research is guided by cognitive culture theory, with culture defined as the knowledge one must acquire to behave adequately in a given social group or context [9,10]. Culture is thus a cognitive phenomenon that is both learned and shared. This approach to culture resolves a number of recurring problems in culture theory in that (a) it provides a satisfying account of the ontology of culture; (b) culture can be located, non-mysteriously, in both social aggregates and individuals; (c) intrasocietal variation as well as sharing of culture can be described; (d) culture as shared knowledge and culture in behavior can be differentiated; and (e) culture can be distinguished from other social-psychological constructs such as values, beliefs, and attitudes [8] (pp. 29–52).

Culture is encoded in cultural models. A cultural model is a skeletal, schematic mental representation of a cultural domain (itself some topic of discourse) that is variably shared with others and socially distributed. A cultural model is made up of the elements that compose a given domain (e.g., “the family”) and how these elements are understood to be related semantically, functionally, and causally within the model. Also encoded within cultural models are prototypes or best exemplars of the domain under consideration. Cultural models can be mundane (e.g., “eating breakfast”) or extraordinary (e.g., “worshipping a deity”). A key feature of cultural models is that they enable us to communicate about a domain more-or-less accurately with others and to make sense of others’ narrative and social practice. Without cultural models, social life would be impossible [11,12].

The study of cultural models has been enhanced by the development of quantitative techniques for examining the degree to which knowledge is shared and distributed and for identifying what knowledge and understanding are most agreed upon [13]. The cultural consensus model is especially valuable in this regard [14]. Cultural consensus analysis is a statistical technique that can be employed once a set of questions has been ethnographically identified that are hypothesized to elicit cultural knowledge in a specific domain. Using a Q-mode form of analysis (i.e., focusing on study subjects as variables) with these data, cultural consensus analysis (CCA) is a latent class model that provides (a) an estimate of the degree of overall sharing of knowledge among respondents about a specific cultural domain; (b) an estimate of the degree to which individual respondents agree with others; and (c) an estimate of how a reasonably knowledgeable individual would respond to that set of questions assessing cultural knowledge within that specific domain (a “cultural best estimate” of the shared knowledge, also referred to as an “answer key” in CCA). CCA has been used to gauge the sharing of knowledge and how that knowledge is distributed in a variety of cultural domains, including ecological knowledge, family life, social support, national identity, and others [15].

CCA itself focuses on shared knowledge and does not address what people do with that knowledge. Given that culture is thought to have a directive and motivating force [16], Dressler [8,17] proposed to extend the cultural consensus model with the development of the concept of “cultural consonance”, defined as the degree to which individuals, in their own behavior, approximate the prototypes for behavior encoded in cultural models. The estimate of the culturally correct answers to a set of questions provided by CCA can be used to create measures of the degree to which individuals correspond to that model in their own behavior or social practice. Cultural consonance is operationalized by the degree to which reported behavior matches the behavior deemed important in the cultural model.

One way of thinking about cultural consonance is that it signals the degree to which individuals have been “culturally successful” in the sense of achieving goals that are collectively valued within society. Dressler [8,17] hypothesized that low cultural consonance, or the condition of being distant from the culturally-valued prototype encoded in a cultural model, would be a stressful experience for a variety of reasons, including the felt distress of being unable to achieve culturally defined goals, and the stigma generated in mundane social interaction when one is not treated with the respect and status accorded the culturally successful, thus leading to poor health outcomes. This hypothesis has been widely replicated in relation to a variety of stress-related health outcomes, including mental health, cardiovascular disease risk, immunocompetence, and body habitus [8,15].

Furthermore, as noted above, we have evidence from prior studies that cultural consonance may be a factor proximate in the causal chain leading to health outcomes [7,8]. At this point in the study of this cultural process, it becomes important to systematically integrate, both theoretically and empirically, the impact it has on health outcomes with other factors, known or suspected to be important influences. One such factor is a sense of personal agency [18,19]. Agency is a particularly important influence to consider since its effect is often considered to be opposed to the influence of culture. That is, if the expectations to follow cultural norms in a given society are strongly enforced, then relatively little leeway would be available for individuals to formulate personal goals and aspirations outside of those norms. At the same time, there are certainly individual differences in the degree to which individuals feel compelled to adhere to those norms. Therefore, in what follows, we suggest that the interplay of culture and agency may be both more subtle and complex than it has been portrayed in earlier work.

### 1.2. A Sense of Personal Agency

A sense of personal agency (referred to by many simply as “agency”) is the individual’s self-evaluation of their capacity for action in the world. Understanding agency in the context of factors that structure behavior, and hence limit agency, has been a focus of study across the social sciences for several decades [18,19]. Not surprisingly, this means that the topic has been approached in several different ways.

In anthropology, agency has been examined with respect to the potential for individuals to act intentionally and pursue their own goals in various cultural settings, although rarely focusing on actual individuals (or “agents”), but rather taking various social types (e.g., “women”, “members of the working class”) and inferring evidence for their agency in relation to specific events or circumstances that are ethnographically observed [19] (pp. 149–150), the focus being on action-in-(cultural)context. Psychologists, on the other hand, have taken a more operational approach to the subject. Bandura [18], in particular, is associated with this work, emphasizing the psychological traits of self-efficacy and locus of control underlying an individual’s capacity for action, (mostly) independent of context.

Following both of these theorists, we define agency first as intentionality, or the purposeful pursuit of a goal. This intent depends on individuals’ belief in their own capacity to act in a given situation, as noted by Bandura [18]. He also argues that agency requires individuals to reflexively evaluate their likelihood of success in achieving those goals. Therefore, any approach to the measurement of agency as an individual difference variable must take at least these two dimensions into account. As Cazzavoni, Fiorini, and Veronese [20] have observed, there are no agreed upon measures of agency in the literature.

In an earlier paper, we introduced a measure of sense of personal agency that combined locus of control and frustration tolerance [7]. Our reasoning followed Bandura [18] in that, first, a measure of the individual’s belief in their own capacity for action was important, and we chose locus of control to represent this. Second, we noted that a belief in one’s own control over the environment that does not include the potential for difficulty or failure in pursuing goals reduces to mere megalomania. We therefore included a measure of frustration tolerance, or the understanding that, at times, achieving a goal can be obstructed and this must be accepted, which captures the reflexive evaluation of agency.

With respect to the role of agency in everyday life, two trends in the literature are useful for understanding the intersection of culture and agency. The first of these is the importance of agency as an influence on status attainment, which in turn can be associated with subjective well-being [21]. The second is the association of a sense of personal agency with subjective well-being, as measured by psychological distress. Thoits [22] argued that there has been insufficient attention devoted to the role of agency in the stress process. She suggests that resistance resources, including coping strategies and social support, which enable individuals to manage stressful events and circumstances, are more effectively mobilized by individuals with a higher sense of their own agency, thus locating agency as a distal influence in a causal sequence leading to subjective well-being.

We combined these perspectives in a study of agency, cultural consonance, and psychological distress in urban Brazil [7]. In that paper, we focused on status attainment in terms of cultural consonance by examining a set of cultural models that define collectively salient adult developmental life goals [23]. These include achieving a reasonable lifestyle, in the sense of culturally defined material consumption and leisure activities; having a network of supportive friends and family; developing a culturally appropriate family life; seeing Brazilian social life in positive terms; and satisfaction with culturally valued educational and occupational opportunities. Using methods of cultural domain analysis (see below), we constructed a measure of cultural consonance with these life goals. We then investigated the relative effects of socioeconomic status, agency, and cultural consonance in life goals on psychological distress. In a hierarchical regression model, both socioeconomic status and agency had substantial associations with psychological distress: higher socioeconomic status and higher agency were associated with lower psychological distress. When cultural consonance was introduced into the model, however, the effects of both socioeconomic status and agency were reduced by about half, and cultural consonance had the strongest association with psychological distress; higher cultural consonance was associated with lower psychological distress (effect size = 0.50) [7].

This suggested a causal sequence in which cultural consonance was the proximate variable to psychological distress, while socioeconomic status and agency were distal or exogenous variables leading to higher cultural consonance. Using path analysis, the results were consistent with this causal model in which cultural consonance mediated the influence of agency and socioeconomic status on subjective well-being [7] (p. 160).

There are two aspects to this model that deserve comment. First, rather than setting culture and agency in opposition, it provides a way to link them in a single process. Second, it is somewhat counterintuitive in that it places culture—or at least one dimension of culture—as a mediator between two psychological variables. As such, it deserves closer scrutiny. This work can thus be viewed in terms of theory building, since the approach we take is novel in the way it links culture and agency.

In order to expand on this work, we carried out a follow-up study in which we collected data on all variables for a randomly selected subset of the original sample on average four years after the initial survey, providing data for a longitudinal test of the causal model. This is important at this point because testing causal models using cross-sectional data can be misleading [24,25]. The results obtained here with longitudinal data will provide a more specific test of the proposed causal model.

### 1.3. The Ethnographic Setting: Urban Brazil

Research was conducted in Ribeirão Preto, Brazil, a city of approximately 700,000 persons located about 300 km north of the city of São Paulo. Ribeirão Preto was established in the late 19th century, serving surrounding coffee plantations as a market center. Since then, the city has grown substantially. The agricultural foundation of the region is still important, although it has now shifted to the cultivation of sugar cane, citrus, and soybeans. The city, while still serving the agricultural industry, has become a regional center for finance, light manufacturing, health care, and especially higher education. It is home to two public universities and several private institutions, as well as three medical programs, a large tertiary care center, and several private hospitals. Throughout Brazil, Ribeirão Preto has a reputation for affluence, although it manifests the same disparities in wealth and social conditions as all of Brazil.

Due to the social and economic disparities, our research has focused on four neighborhoods varying along these dimensions for both ethnographic and survey research; furthermore, we have carried out several research projects that span a period of over 30 years [8]. The first and least affluent neighborhood began as what in Brazil is referred to as a *favela* or “squatter settlement”. These *bairros* (neighborhoods) begin when a number of people “invade” unused land and build homes out of whatever materials can be found. The neighborhoods can quickly assume a permanence as enterprising residents tap into passing municipal water and electrical services and organize socially and politically to advocate for the community. Residents are generally unstably employed in unskilled manual occupations such as agricultural and construction labor for men and as domestic servants for women. Educational attainment is correspondingly low. This particular neighborhood underwent a substantial transformation about 30 years ago when the municipality removed the population from their original site and moved them to a housing complex of two-room cinderblock houses. Due to governmental economic policies in the late 1990s and early 2000s, the community saw small but significant increases in household incomes; however, it still has the lowest household incomes and educational attainment of the four neighborhoods and retains an unsavory reputation for drug trafficking.

The second neighborhood is somewhat higher in household income and educational attainment. It began as a *conjunto habitacional* (housing development). Such complexes are established in various ways, frequently as a cooperative venture of either a public or private financial institution and a developer. Often a lottery is held, in which individuals qualify for favorable financing on houses by demonstrating stability in employment. Houses are again fairly simple four- to five-room cinderblock constructions, although these neighborhoods take on a distinctive appearance as residents add garden walls, new rooms, and even second floors. After 30 years of existence, this development now has a commercial area with shops and a supermarket. Residents are stably employed as skilled labor in factories, as technicians, and in occupations such as nurse’s aid or store clerk.

The third neighborhood is a venerable middle class community that dates nearly to the founding of the city. It boasts its own Catholic church in front of a large *praça* (plaza), along with a major commercial area. The streets are cobblestone and houses present seamless walls to the street. Founded by Italian immigrants in the early 20th century, in the imagination of the city, this neighborhood is a repository of European-Brazilian values, so much so that even a nationally well-received book of poetry about growing up there has been published. The neighborhood has somewhat higher household incomes and educational attainment than the second neighborhood, although the differences are diminishing as the housing stock ages and newer developments are more easily reached, given the increase in individual automobile ownership. Residents of the third community tend to be employed in highly skilled occupations, including nurses, teachers, and managers of larger businesses.

The fourth and final neighborhood is a *condomínio* (gated community). Adjacent to one of the universities, the community residents include doctors, lawyers, university professors, and the owners of large businesses and manufacturing concerns. Contemporary spacious houses sit on expansive lots. It has the highest household incomes and educational levels of the four neighborhoods.

Our research in Ribeirão Preto over a period of 30 years has focused on cultural models that structure adult life goals in everyday life [23]. While our sampling of this sphere is certainly not exhaustive, the domains we have selected, including lifestyle, social support, family life, Brazilian identity, and occupational and educational aspirations, capture core objectives for individuals building a life. These domains were selected for investigation because of the frequency with which these topics arise in mundane conversation, and cultural domain analysis was used to closely examine each domain. Free lists were used first to elicit the terms and phrases that populate and structure the domain. In free list interviews, respondents were prompted to provide terms and phrases that describe the composition of the domain (e.g., for national identity, respondents were asked “what is it that makes a Brazilian, Brazilian?”). After collating the free lists and reconciling alternate phrasing and synonyms, terms and phrases that were employed by at least 10% of the sample interviewed (n = 43) were then retained for further analysis of that cultural domain. These lists were in the range of 20–30 terms, depending on the domain. So, for example, the domain of family life included items referring to the emotional climate of the family, as well as to its structure and organization, and to characteristics that could threaten the family (e.g., egoism). Then, the internal structure of each domain was analyzed using unconstrained pile sorts and nonmetric multidimensional scaling. One dimension structuring all of the domains stood out: Respondents tended to group items in terms of their relative importance in creating the domain configuration. Within the domain of lifestyle, for example, a central dimension along which elements of the domain were grouped was their relative importance “*para ter uma vida*” (in order to have a life).

At that point, another sample (n = 66) rated or ranked items in each domain according to their importance. These data were employed in a cultural consensus analysis to determine (a) if there was sufficient agreement among respondents to make reasonable the inference that they shared a cultural model regarding the importance of items and (b) how that agreement was distributed. We found that for each domain, there was strong enough consensus to infer a shared cultural model for that domain. Furthermore, the variation in that consensus was examined in relation to neighborhood, gender, and age. While there was some slight variation (e.g., women agreed more strongly about the importance of characteristics of family life than men; younger people agreed more about the importance of specific sources of social support than older people), the evidence supported a single cultural model in each domain cutting across neighborhood, age, and gender [26].

Measures of cultural consonance with each model were then developed for use in social survey research [26]. Scale questions were generated within each model that assessed whether the respondent had successfully incorporated culturally defined important items into their own lives. This was relatively straightforward with lifestyle and social support (i.e., did people own material goods and participate in leisure activities identified as important in the model?; did people turn to culturally salient sources of support such as family, friends, and co-workers in response to problems?). Questions regarding family life, national identity, and vocation were somewhat more complicated and required Likert-response items capturing the respondent’s perceptions of that domain (i.e., did people see their families as close, supportive, and well-organized?). Unlike the cultural consensus regarding the importance of items within each domain, the measures of cultural consonance in each domain showed considerable variation within the sample, especially across the four neighborhoods differing in socioeconomic status. In other words, Brazilians of different social status agreed strongly about important goals in adult life; they were less uniformly successful in achieving those goals. Furthermore, the higher an individual’s cultural consonance in each domain, the lower their blood pressure, reported psychological distress, immune system activation, and body mass, controlling for age, gender, socioeconomic status, and other covariates relevant to each specific outcome variable [8,15].

Finally, we found that cultural consonance with each cultural model was correlated, with all five measures loading a single principal component. This led us to hypothesize that these five cultural models were cognitively organized in terms of a more abstract cultural domain of *metas na vida* (goals in life). A free list of life goals elicited items associated with each of the five specific domains. We then found cultural consensus regarding the importance of life goals, thus lending evidence to our inference that the principal component combining the five measures of cultural consonance indeed assessed cultural consonance in life goals. Furthermore, low cultural consonance in the composite measure of life goals was associated with higher psychological distress [23].

These studies demonstrate the salience of these cultural models in structuring the more general domain of life goals, as well as the importance of cultural consonance with these models in everyday life in Brazil. These are broadly shared aspirations, and if an individual is not successful in achieving these goals—that is, if they have low cultural consonance—their risk of adverse health outcomes increases. Hence, the structuring influence of culture in Brazilian society is clearly evident.

At the same time, there is, in Brazilian society, a tradition of skirting the constraints of social life that indicates the cultural salience of personal agency. This is the practice of *o jeitinho*. The term *jeito* can be simply translated as “way, manner, or knack”. In the diminutive form, however, it refers specifically to a way of bending or breaking normal rules of social life in order to achieve a goal. A classic example of *o jeitinho* is of someone going to a government agency to transact some business (e.g., to renew a driver’s license). Brazilians are required to present a number of official documents in such cases, and frequently one may be missing or forgotten. After waiting a significant amount of time and not wanting to return home, then return to the agency, and then wait again, a person might ask “*me dá um jeitinho*?”, which could translate into English roughly as “would you give me a break?” While this is a simple example, *o jeitinho* can be employed in virtually any setting (e.g., trying to get a court case heard or a surgery scheduled). The salience of *o jeitinho* in Brazil can be traced to historical power dynamics in the colonial plantation system and the relative lack of power of those occupying any position in the social hierarchy below that of the landowner. While it is a controversial practice in Brazil since it does in essence require a social transgression, there is nonetheless the recognition that for many people in some situations, *o jeitinho* is a necessary way of adapting in everyday life [7,27].

It has been suggested that *o jeitinho* is a stable behavioral trait in individuals that is associated with basic personality type [28]. For our purposes here, we focus on it more as a commonly understood cultural theme, perhaps a cultural model (although we have no specific data on that), which codes for a sense a personal agency. Put differently, *o jeitinho* recognizes and defines the option of moving outside expected cultural models (or norms) in order to achieve one’s goals. Hence, it reinforces the development of a sense of personal agency for individuals in Brazilian social life.

As we noted earlier, while many authors in the social sciences see culture and agency as opposed, based on our previous findings, we see no reason why one’s sense of agency could not be employed to achieve the goals and aspirations encoded in cultural models, thus leading to higher cultural consonance (noted by Ortner [19] as well). Our previous study confirmed this, which led us to posit a causal model in which higher socioeconomic status and personal agency lead to higher cultural consonance, which in turn leads to lower psychological distress [7]. We now present a further test of this hypothesis using longitudinal data.

## 2. Materials and Methods

Research was carried out in the city Ribeirão Preto, Brazil. The research was approved by the Institutional Review Board for the Protection of Human Subjects of The University of Alabama (Protocol #10-22-ME) and the *Comité de Ética de Pesquisa* (Research Ethics Committee) of the University of São Paulo-Ribeirão Preto (Protocol #4384/2011). All respondents provided written informed consent.

### 2.1. Sampling

As noted above, all data collection took place in each of four neighborhoods stratified by economic status. The original cultural domain analyses for testing cultural consensus in each of the five domains was carried out in 2001, and four of the five scales of cultural consonance were then developed [26]. In the study reported here, begun in 2011, we replicated the main parts of the cultural domain analysis for the four domains examined earlier and carried out a complete domain analysis for the domain of occupational and educational aspirations, which was then added with a convenience sample of 40 persons (10 from each neighborhood). Subjects were recruited in order to resemble the original sample from 2011 in terms of age and gender, with each economic stratum represented [29].

The original cross-sectional social survey sample consisted of 477 respondents. Occupied addresses were randomly selected using maps of each neighborhood. These households were then visited by research assistants who explained the research and solicited one adult over the age of 18 to participate. Combining refusals and households that could not be contacted, the final sample represented a response rate of 31%.

A subsample of 100 individuals (25 from each neighborhood) was randomly selected for follow-up, and interviews were completed with 64 respondents. The average time between initial interviews and follow-up interviews was 4 years, with the majority of respondents interviewed between 2 and 6 years later. Interviews were conducted by Brazilian research assistants trained by the authors.

### 2.2. Measurement

For each measurement in the model, except for age and gender, standardized scores for two or more variables were combined to create scales. In the follow-up sample (n = 64), scores were standardized using the means and standard deviations of the original sample (n = 477). This insured that comparisons over time were in the same metric.

Dependent variable—The dependent variable is psychological distress, measured by a principal components score combining a Brazilian translation of the Center for Epidemiologic Studies-Depression Scale (CES-D) [30] and our own translation of Cohen’s Perceived Stress Scale (PSS) [31]. We have used both scales in several studies, and the reliabilities average around 0.90 for the CES-D and 0.80 for the PSS.

Covariates—Covariates include age (in years) and gender (female = 0; male = 1).

Socioeconomic status—Socioeconomic status (SES) is a standardized score combining years of education of the respondent and household income.

Cultural consonance in life goals—As noted above, the measurement of cultural consonance involves a two-stage process, the first of which is the cultural domain analysis and verification of a cultural model in each domain, the second of which is the development of scales of cultural consonance employing the results of the CCA in each domain. All scales of cultural consonance (with the exception of occupational and educational aspirations) were developed in 2001 [26]. In the study presented here, we replicated most of the cultural domain analysis in each domain (carrying out a full domain analysis for occupational and educational aspirations) with the convenience sample of n = 40 described above. We did not fully replicate the free list interviews but asked instead if there were any items that should be added to those items already employed (there were not). Then, the sorting tasks and rating and ranking tasks were replicated. We again found cultural consensus in each domain (including occupational and educational aspirations). The “cultural best estimates” (also referred to as “answer keys” in CCA) of the consensus ratings or rankings of the importance of the items for three out of the four consensus models comparing the 2001 and 2011 results were correlated at r > 0.90; the only exception was the cultural consensus models of social support, which correlated at r = 0.84 [29]. This analysis confirms the validity of continuing to use the cultural consonance scales developed in 2001 for our research ten years later.

Cultural consonance in each domain was measured as follows:

- Lifestyle—Respondents reported ownership of culturally salient material goods (14 items) and engaging in culturally salient leisure activities (7 items); self-reports were summed (alpha = 0.73). Sample material goods include a home, a car, kitchen appliances, furniture, and a computer. Sample leisure activities include time for rest and study, practicing sports, and going out with family or friends.
- Social support—Respondents ranked 7 potential supporters in the order in which they personally would seek support from them for each of 9 problems. Each individual’s correlation with the consensus ranking was calculated to measure consonance. Problems ranged from mundane difficulties (needing a ride to work) to more stressful circumstances (such as family problems). Potential sources of support included family and friends, church members, and professionals.
- Family life—Respondents reported perceptions of their family in terms defined by the consensus model on an 18-item Likert-response scale (alpha = 0.91). Characteristics from the cultural consensus model describing families included love and support within the family as well as family structure and organization. Items were weighted by their rank order in the cultural consensus model.
- National identity—Identification with positive characteristics of Brazilians was assessed with an 8-item Likert-response scale (alpha = 0.60). These included being sociable and avoiding practices such as *o jeitinho*.
- Education/occupation—Respondents rated their satisfaction with culturally-salient occupational and educational opportunities (alpha = 0.81). These included having the opportunities for formal study and receiving recognition and advancement within the workplace.

A principal component score combining these five variables that explained 44% of the variance was employed as a measure of cultural consonance in life goals. The principal component analysis returned a single component with an eigenvalue > 1.0. Additionally, other analyses with fewer restrictive assumptions (i.e., nonmetric multidimensional scaling and cluster analysis) are consistent with there being a latent factor of cultural consonance in life goals. We employ principal component analysis because it is a useful method of combining the five measures in a weighted average. (See previous publications [8,26] for more detail on the measurement.)

Sense of personal agency—The scale of a sense of personal agency combines measures of locus of control and frustration tolerance. We used our own translation of Coreil and Marshall’s [32] Locus of Control Scale designed for cross-cultural research. Scale items include “When I have a problem, I do something to resolve it”. We have used this scale in a number of studies, and it has acceptable reliability (alpha > 0.75). For frustration tolerance, we translated Harrington’s Frustration Discomfort Scale [33], which also displays acceptable reliability (alpha = 0.92). It includes items such as “I can’t stand to wait for things I would like to have now”. These two scales are positively correlated (r = 0.44). They were standardized and summed to form a measure of sense of personal agency.

## 3. Results

Table 1 presents descriptive statistics for all variables for the original survey sample (n = 477) and the follow-up subsample (n = 64) measurements at Time 1 and Time 2. Tests of statistical significance (using single sample *t*-tests comparing subsample means to the original sample means) were carried out to assess the goodness-of-fit of the follow-up sample to the original survey sample at Time 1. The only difference was a somewhat lower SES. This was due primarily to a smaller number of households in the wealthiest neighborhood agreeing to be interviewed at follow-up than in other neighborhoods.

With respect to differences between Time 1 and Time 2, the respondents were obviously older at Time 2. SES increased from Time 1 to Time 2. A detailed inspection of the responses showed that this was due to seven respondents completing advanced education or training in the interval between the interviews and receiving a somewhat higher income. Finally, expressions of a sense of personal agency declined from Time 1 to Time 2.

Table 2 presents bivariate correlations among all variables included in the model.

**Figure 1:**
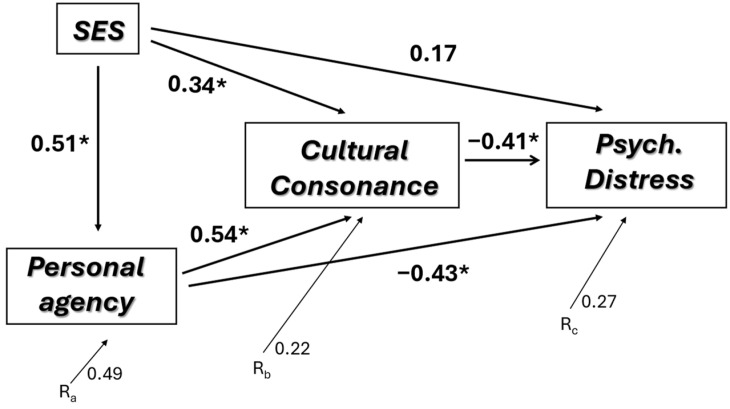
Path model of factors influencing psychological distress (Time 1 data only). * *p* < 0.05.

Figure 1 presents a simple path analysis [34,35] with SES and personal agency as exogenous variables and cultural consonance as the endogenous, mediating variable leading to psychological distress (standardized path coefficients are reported). The path analysis replicates the cross-sectional analysis we presented earlier [7], using only the follow-up subsample at Time 1. We present this model here to replicate our prior findings in this subsample [7] and to present the cross-sectional baseline of our findings. SES has a substantial association with personal agency. Both variables also have substantial influences on cultural consonance, which in turn has a substantial association with psychological distress. Any direct influence of SES on psychological distress disappears in this analysis, while personal agency has a substantial direct influence on the outcome variable. (Note that age and gender are controlled for in these analyses but were not assigned causal paths.) 

**Figure 2:**
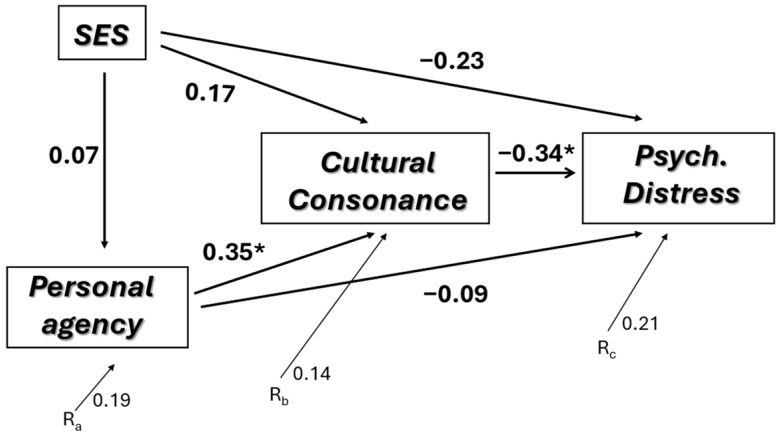
Path model of factors influencing psychological distress (change in Time 1 to Time 2). * *p* < 0.05.

Figure 2 presents this path analysis using longitudinal data. In order to focus exclusively on change over time, the model recommended by Cohen and Cohen [36] was employed. In this model, in the regression equations estimating the path coefficients, Time 1 variables were entered first a as block; then, Time 2 variables were entered to estimate the path coefficients associated with change over time. In this model, residual values in Time 2 variables represent change over time in that the values of all Time 1 variables have been controlled for. Put differently, the only variation in Time 2 variables left after controlling for Time 1 variables is that associated with change over time (discounting, of course, possible effects of unmeasured variables). Therefore, covariation among Time 2 variables is not influenced by Time 1 values.

In the model presented in Figure 2, the direct effect of SES on personal agency disappears, while personal agency continues to have a significant effect on cultural consonance; however, the direct effect of personal agency on psychological distress disappears. SES has no direct effect on psychological distress, while the effect of cultural consonance on psychological distress remains relatively large. This causal model accounts for 61.7% of the variance in psychological distress.

Due to the small sample size, influential case analysis was carried out by calculating studentized deleted residuals and leverage values for the path analysis presented in Figure 2. All residuals were within a range of −2.9 to +2.9, and there were no large leverage values. Additionally, tolerance values (or the related variance inflation factor) were all within normal limits.

## 4. Discussion

This longitudinal test of a path model, including cultural consonance as a proximate variable in the causal sequence leading to psychological distress, with socioeconomic status and personal agency as distal, exogenous variables, overall replicates our previous findings based on a cross-sectional analysis of a larger sample [7]. It is worth noting, too, that our findings are congruent with the longitudinal model tested by Hitlin and associates [21].

These results are also consistent with other studies in which cultural consonance appeared to play a mediating role for perceived stress [37], socioeconomic status [38], and gene-environment interaction [39] in relation to psychological distress, although these were all cross-sectional studies. Finally, other investigators have replicated these findings in a longitudinal model [40] in research in Bolivia.

The major difference between the cross-sectional and longitudinal findings involves the direct effects of socioeconomic status on personal agency and cultural consonance, and the direct effects of socioeconomic status and personal agency on psychological distress. In the cross-sectional analysis of these data, socioeconomic status had a substantial positive influence on personal agency. Then, both socioeconomic status and personal agency were strongly positively associated with cultural consonance, which in turn had a substantial association with psychological distress. Personal agency also had a large direct association with psychological distress. In bivariate analyses, socioeconomic status was inversely associated with psychological distress (as expected), but when cultural consonance was entered into the model, this association disappeared.

These results changed substantially when the longitudinal data were analyzed. First, the effect of socioeconomic status on personal agency was not evident in the longitudinal study, nor was the effect of socioeconomic status on cultural consonance or psychological distress. Second, the direct effect of personal agency on psychological distress disappeared; however, change over time in personal agency was moderately strongly associated with higher cultural consonance. Third, change in cultural consonance over time had a moderately strong association with change over time in psychological distress.

The lack of an effect of socioeconomic status on personal agency or cultural consonance is not too surprising, in that socioeconomic status likely changes little for most people over this time span. As noted, for seven individuals in the follow-up sample, there was an increase in socioeconomic status as they completed their educations and their incomes increased, leading to a significant difference between Time 1 and Time 2; however, this difference could certainly be due in part to the small size of the follow-up sample. The number of individuals enjoying this socioeconomic mobility is likely to be proportionately smaller in a larger follow-up sample. It is interesting to note that in the longitudinal data, socioeconomic status at Time 1 continued to have a substantial effect on personal agency at Time 2, suggesting that the long-term effects of socioeconomic status may be more important than short-term fluctuations (which is what our model in Figure 2 assesses). When the model in Figure 2 is replicated employing only the Time 1 measure of socioeconomic status, there is a distinct causal pathway of higher SES leading to higher personal agency (beta = 0.39), along with higher personal agency leading to higher cultural consonance (beta = 0.33), and higher cultural consonance leading to lower psychological distress (beta = −0.37). This suggests that the primary effect of socioeconomic status in this process is to lay the groundwork for the development of a sense of personal agency, which, as it develops, leads to the achievement of higher cultural consonance. In future research, examining this causal sequence, it may be useful to focus on socioeconomic status in the long-term rather than the short-term.

The decline in personal agency from Time 1 to Time 2 could be interpreted in a number of ways. In examining this decline more closely, it was concentrated in locus of control, which significantly declined between the time periods (*p* < 0.01), meaning that there was a mean shift from a higher internal locus of control to a higher external locus of control. Frustration tolerance exhibited no such change. Accounting for this change can only be a matter of informed speculation, but the decade of the 2010s, during which these data were collected, was one of substantial change and turmoil in Brazil. During the initial decade of the 21st century, Brazil was governed by the center-left *Partida dos Trabalhadores* (Worker’s Party) and its charismatic president Luiz Ignácio Lula da Silva (“Lula”). During his two terms in office, Brazil enjoyed a period in which income inequality declined and household incomes increased in the poorest neighborhoods (while still having the lowest average household incomes), in part as a function of the moderation of inflation (which actually began to moderate in the earlier administration of Fernando Henrique Cardoso) and an increase in social services. Lula was replaced by his protégé Dilma Rousseff, a technocratic economist who lacked his charisma. Higher inflation returned during the 2010s, and Rousseff was vulnerable to attacks by political opponents, ultimately leading to her impeachment and large-scale disruption in the political system, which in turn led to wide-spread protests and strikes.

Pincheiro-Macado and Scalco [41] argue that the result of this decades-long process was first, under Lula’s administration, to open up the potential for people who were less well-off to at least have hope that they might increase their cultural consonance. With the worsening economy under Rousseff and the subsequent political turmoil, this hope proved to many to be illusory. It may be, then, that the decline in a sense of personal agency, driven mostly by a decline in internal locus of control, reflects these larger political-economic forces in the everyday lives of people when they saw the hope for valued gains lost. Again, this is informed speculation regarding this finding and awaits further examination.

The principal finding of this analysis is, however, the continuing evidence for the mediating role of cultural consonance in accounting for psychological distress. Even though there was a mean decline in personal agency, for those individuals whose sense of their own agency increased over the period between Time 1 and Time 2, their cultural consonance in life goals also increased, leading to lower psychological distress. Three implications of this finding stand out. First, as we noted earlier, in discussions of culture and agency, especially in anthropology, the initial assumption seems to be that culture and agency are opposed—that the exercise of one’s agency is meant to escape the constraints of cultural norms. Many of these discussions of agency do, however, focus on groups who, in some societies, are subject to culturally sanctioned constraints, such as women or ethnic minorities. There certainly are efforts to exercise agency to break out of such constraints. Even so, however, there are collectively valued goals in society, the pursuit of which is recognized as achieving cultural success and, hence, a measure of higher social status. It is not surprising that individuals would direct their sense of personal agency toward achieving these collective goals. Indeed, it is entirely likely that personal agency is focused in both ways by the same individuals.

Second, the role of cultural consonance as a mediating variable between two psychological variables is novel and, given current thinking, somewhat counterintuitive. For many scholars, culture is considered to be an aggregate variable, and its influence is in how it provides the context within which individual-level variables such as motivation and personality directly influence subjective well-being. The notion that culture could be an outcome of motivation and personality, or that it would have direct effects on psychological outcomes, is rarely encountered. A strength of cognitive culture theory is that it enables the investigator to see culture as, non-mysteriously, both an aggregate- and an individual-level variable. At the aggregate level, culture is shared knowledge and does not correspond precisely to any individual’s knowledge. At the individual level, culture represents both what knowledge individuals share with others, and it represents how individuals correspond to cultural consensus models in their own social practice. This theoretical orientation thus enables us to see culture as a broad environment of meaning, which in turn provides the context for culture to be incorporated into individual understanding and behavior. A more nuanced view of culture along multiple dimensions is empirically useful since cultural consonance appears to have the most impact.

Third, and perhaps most generally, these findings point to the importance of including cultural variables in the study of individual differences in status attainment and subjective well-being. Many studies continue to rely on simple measures of status attainment based on traditional indicators such as education or occupational status. The results presented here can parse this process more finely. Focusing on cultural consonance provides a more “experience near” rendering of how individuals in a specific cultural context focus their energies on achieving those things that are valued in society, and of the implications of achieving or not achieving cultural success.

It is worth noting, too, that even with simpler forms of analysis, such as a regression model in which socioeconomic status, personal agency, and cultural consonance are treated as all having direct effects on psychological distress, the results are consistent with cultural consonance as a proximate mediator of other variables, just as we observed in our original cross-sectional study [7]. Once cultural consonance enters the equation, the effects of the other variables are substantially reduced, supporting the inference that cultural consonance is proximate in a causal sequence. As we noted above, evidence is converging across several studies to support this inference.

While it could be argued that cultural consonance is a “measure of stress”, we prefer to think of cultural consonance as a basic part of human social life. Low cultural consonance is certainly a stressful experience, but thinking of it exclusively in these terms may obscure other important factors. One of these is captured by Erving Goffman’s phrase: “the presentation of self in everyday life” [42]. Cultural consonance, from readily observable aspects to one’s display of shared knowledge, projects into mundane social interaction one’s position in a cultural space defined by that knowledge and by social practice [43]. The closer the individual is to the prototype defined, in the example presented here, by successfully achieving adult life goals, the higher the status accorded that individual. Conversely, persons distant from that prototype see themselves and, importantly, are seen by others not to have achieved that level of cultural success. This then results in individuals not being accorded the basic respect of being a person in that society and social setting. As we observed in our cultural domain analysis of life goals, the cultural semantic space defined by these goals literally revolved around two goals in the multidimensional scaling plot: achieving respect and recognition [23] (p. 51). The stigma of having these withheld in mundane social interaction leads to distress.

There are a number of limitations of this study, the first and most important being sample size. We had hoped to obtain a larger follow-up sample, but logistical factors limited our ability to do so. The effect sizes observed in research on cultural consonance are such that even this small sample was not dramatically under-powered, and the influential case analysis indicated no problem with specific cases overly affecting the findings. Given also that the follow-up sample only differed slightly from the much larger sample from which it was derived, these findings cannot easily be dismissed because of sampling error.

While not precisely a limitation, some readers may be concerned about the strong correlations among the variables leading to unreliability in the estimates of the parameters. The variable tolerances in all regression equations estimating the path coefficients were acceptable (>0.25).

For some, a limitation of this research might be our use of a relatively simple classic path analysis rather than a full structural equation model, including confirmatory factor analysis for measurements [44], although it should be recalled that path analysis is a special case of the larger family of structural equation models [35]. Our decision in this regard was again a function of the sample size, although measurement issues were also a concern. Our sample size seemed too small to estimate all of the parameters output by a structural equation model with confirmatory factor analysis. Additionally, with our focus on constructing a particular measure of cultural consonance, based on cultural domain analysis, we believed it to be a better strategy in this paper to employ the simpler analysis. Again, more complex analytic models can be used in future research, including combining causal and multilevel models.

We have specified a fairly simple model, employing only three major independent variables plus two additional covariates. Certainly, there are other important factors involved. Nevertheless, the longitudinal model accounts for more than 60% of the variance in psychological distress, suggesting that it is well-specified.

Finally, we have discussed these findings in terms of causality, as though it were a straightforward notion. Even though we have demonstrated covariation through time among these variables, our research design still cannot reject the possibility of reciprocal causation, particularly feedback loops connecting cultural consonance and psychological distress. Taking into account these kinds of relationships will require a more complex research design with multiple data collection points through time [25].

Despite these limitations, the results presented here offer a longitudinal replication of a previously proposed model linking social, psychological, and cultural variables in accounting for subjective well-being. The model is somewhat novel in the way the interplay among the variables is specified. Future research should examine this further.

## Figures and Tables

**Table 1:** Descriptive statistics for the original survey sample, the follow-up subsample at Time 1, and the follow-up subsample at Time 2.

Variables:	Original Sample (n = 477)	Follow-Up Sample Time 1 (n = 64)	Follow-Up Sample Time 2 (n = 64)
Psychological Distress	−0.01 (±0.99)	−0.10 (±1.91)	−0.04 (±1.04)
Age	48.2 (±14.7)	50.4 (±14.2)	54.2 (±14.3) ^b^
Gender (% male)	34.0	27.0	27.0
SES *	0.30 (±0.77)	−0.16 (±0.66) ^a^	−0.08 (±0.98) ^b^
Cultural Consonance	0.00 (±1.00)	0.02 (±0.99)	−0.06 (±1.71)
Personal Agency	0.00 (±1.00)	−0.06 (±1.09)	−0.32 (±1.12) ^b^

* SES = socioeconomic status; ^a^ *p* < 0.05 (as goodness-of-fit to the original overall sample, using single sample *t*-tests comparing subsample means to the original sample means); ^b^ *p* < 0.05 (difference between Time 1 and Time 2 within the follow-up sample).

**Table 2:** Correlation matrix of all variables.

	Distress 1	Distress 2	Age (Time 1)	Gender (Time 1)	SES1	SES2	Cultural Consonance 1	Cultural Consonance 2	Agency 1	Agency 2
Distress 1	1	0.707 **	−0.142	−0.206	−0.171	−0.294 *	−0.637 **	−0.583 **	−0.638 **	−0.462 **
Distress 2	0.707 **	1	0.017	−0.201	−0.250 *	−0.400 **	−0.618 **	−0.668 **	−0.595 **	−0.523 **
Age (Time 1)	−0.142	0.017	1	0.103	−0.250 *	−0.250 *	0.036	0.048	−0.078	−0.172
Gender (Time 1)	−0.206	−0.201	0.103	1	0.013	0.065	0.099	0.148	0.052	−0.021
SES 1	−0.283 *	−0.327 **	−0.175	0.055	1	0.866 **	0.558 **	0.517 **	0.510 **	0.641 **
SES 2	−0.230 *	−0.374 **	−0.256 *	0.012	0.850 **	1	0.603 **	0.543 **	0.496 **	0.571 **
Cultural consonance 1	−0.637 **	−0.618 **	0.036	0.099	0.559 **	0.564 **	1	0.831 **	0.707 **	0.644 **
Cultural consonance 2	−0.583 **	−0.668 **	0.048	0.148	0.492 **	0.521 **	0.831 **	1	0.664 **	0.686 **
Agency 1	−0.638 **	−0.595 **	−0.078	0.052	0.488 **	0.537 **	0.707 **	0.664 **	1	0.755 **
Agency 2	−0.462 **	−0.523 **	−0.172	−0.021	0.559 **	0.580 **	0.644 **	0.686 **	0.755 **	1

* *p* < 0.05. ** *p* < 0.01

## Data Availability

Data are contained within the article.

## References

[1] Ortner S.B. (2005). Subjectivity and cultural critique. Anthropol. Theory.

[2] Sewell W.H. (1992). A theory of structure: Duality, agency, and transformation. Am. J. Sociol..

[3] Archer M.S. (2003). Structure, Agency and the Internal Conversation.

[4] Bourdieu P. (1984). Distinction: A Social Critique of the Judgement of Taste.

[5] Giddens A. (1979). Central Problems in Social Theory: Action, Structure, and Contradiction in Social Analysis.

[6] Ahearn L.M. (1999). Agency. J. Linguist. Anthropol..

[7] Dressler W.W., Balieiro M.C., dos Santos J.E. (2019). Agency, Cultural Consonance and Depressive Symptoms: A Brazilian Example. Ethos.

[8] Dressler W.W. (2018). Culture and the Individual: Theory and Method of Cultural Consonance.

[9] Goodenough W.H. (1956). Cultural anthropology and linguistics. Phila. Anthropol. Soc. Bull..

[10] Goodenough W., Levinson D., Ember M. (1996). Culture. Encyclopedia of Cultural Anthropology.

[11] D’Andrade R. (1995). The Development of Cognitive Anthropology.

[12] Bennardo G., Bennardo G., de Munck V.C., Chrisomalis S. (2024). Cultural Model Theory: Culture, Mind, and Knowledge. Culture in and out of Mind: Cultural Model Theory.

[13] Weller S.C. (2007). Cultural Consensus Theory: Applications and Frequently Asked Questions. Field Methods.

[14] Romney A.K., Weller S.C., Batchelder W.H. (1986). Culture as consensus: A theory of culture and informant accuracy. Am. Anthropol..

[15] Dressler W.W. (2020). Cultural Consensus and Cultural Consonance: Advancing a Cognitive Theory of Culture. Field Methods.

[16] D’Andrade R., Schweder R.A., LeVine R.A. (1984). Cultural Meaning Systems. Culture Theory: Essays on Mind, Self, and Emotion.

[17] Dressler W.W. (1996). Using cultural consensus analysis to develop a measurement: A Brazilian example. Cult. Anthropol. Methods.

[18] Bandura A. (2018). Toward a psychology of human agency: Pathways and reflections. Perspect. Psychol. Sci..

[19] Ortner S.B. (2006). Anthropology and Social Theory: Culture, Power, and the Acting Subject.

[20] Cavazzoni F., Fiorini A., Veronese G. (2022). How Do We Assess How Agentic We Are? A Literature Review of Existing Instruments to Evaluate and Measure Individuals’ Agency. Soc. Indic. Res..

[21] Hitlin S., Erickson L.D., Brown J.S. (2015). Agency and Mental Health: A Transition to Adulthood Paradox. Soc. Ment. Health.

[22] Thoits P.A. (2006). Personal Agency in the Stress Process. J. Health Soc. Behav..

[23] Dressler W.W., Balieiro M.C., dos Santos J.E. (2017). Cultural Consonance in Life Goals and Depressive Symptoms in Urban Brazil. J. Anthropol. Res..

[24] Jose P.E. (2016). The Merits of Using Longitudinal Mediation. Educ. Psychol..

[25] Preacher K.J. (2015). Advances in Mediation Analysis: A Survey and Synthesis of New Developments. Annu. Rev. Psychol..

[26] Dressler W.W., Borges C.D., Balieiro M.C., dos Santos J.E. (2005). Measuring Cultural Consonance: Examples with Special Reference to Measurement Theory in Anthropology. Field Methods.

[27] Barbosa L. (1992). O Jeitinho Brasileiro.

[28] Miura M.A., Pilati R., Milfont T.L., Ferreira M.C., Fischer R. (2019). Between simpatia and malandragem: Brazilian jeitinho as an individual difference variable. PLoS ONE.

[29] Dressler W.W., Balieiro M.C., dos Santos J.E. (2015). Finding Culture Change in the Second Factor Stability and Change in Cultural Consensus and Residual Agreement. Field Methods.

[30] da Silveira D.X., Jorge M.R. (2002). Reliability and factor structure of the Brazilian version of the Center for Epidemiologic Studies-Depression. Psychol. Rep..

[31] Cohen S., Karmack T., Mermelstein R. (1983). A global measure of perceived stress. J. Health Soc. Behav..

[32] Coreil J., Marshall P. (1982). Locus of illness control: A cross-cultural study. Hum. Organ..

[33] Harrington N. (2005). The Frustration Discomfort Scale: Development and psychometric properties. Clin. Psychol. Psychother..

[34] Hadden K., DeWalt B.R. (1974). Path analysis: Some anthropological examples. Ethnology.

[35] Lleras C., Kempf-Leonard K. (2005). Path analysis. Encyclopedia of Social Measurement.

[36] Cohen J., Cohen P. (1975). Applied Multiple Regression/Correlation Analysis for the Behavioral Sciences.

[37] Balieiro M.C., dos Santos M.A., dos Santos J.E., dos Dressler W.W. (2011). Does perceived stress mediate the effect of cultural consonance on depression?. Transcult. Psychiatry.

[38] Dressler W.W., Balieiro M.C., Ribeiro R.P., dos Santos J.E. (2015). Culture as a mediator of health disparities: Cultural consonance, social class, and health. Ann. Anthropol. Pract..

[39] Dressler W.W., Balieiro M.C., Ferreira de Araújo L., Silva W.A., dos Santos J.E. (2016). Culture as a mediator of gene-environment interaction: Cultural consonance, childhood adversity, a 2A serotonin receptor polymorphism, and depression in urban Brazil. Soc. Sci. Med..

[40] Reyes-García V., Gravlee C., McDade T., Huanca T., Leonard W., Tanner S. (2010). Cultural Consonance and Psychological Well-Being. Estimates Using Longitudinal Data from an Amazonian Society. Cult. Med. Psychiatry.

[41] Pinheiro-Machado R., Scalco L.M. (2020). From hope to hate: The rise of conservative subjectivity in Brazil. HAU J. Ethnogr. Theory.

[42] Goffman E. (1959). The Presentation of Self in Everyday Life.

[43] Dressler W.W., Balieiro M.C., dos Santos J.E. (2023). Distance from a Cultural Prototype and Psychological Distress in Urban Brazil: A Model. J. Cogn. Cult..

[44] MacCallum R.C., Austin J.T. (2000). Applications of structural equation modeling in psychological research. Annu. Rev. Psychol..

